# Real-World Evidence Analysis of a Hybrid Closed-Loop System

**DOI:** 10.1177/19322968231185348

**Published:** 2023-07-08

**Authors:** Heba Alwan, Malgorzata E. Wilinska, Yue Ruan, Julien Da Silva, Roman Hovorka

**Affiliations:** 1 Wellcome-MRC Institute of Metabolic Science, University of Cambridge, Cambridge, UK; 2 Institute of Primary Health Care (BIHAM), University of Bern, Bern, Switzerland; 3 Ypsomed AG, Burgdorf, Switzerland

**Keywords:** type 1 diabetes, artificial pancreas, real-world evidence, closed-loop systems

## Abstract

**Background:**

We analyzed real-world evidence to assess the performance of the mylife CamAPS FX hybrid closed-loop system.

**Methods:**

Users from 15 countries across different age groups who used the system between May 9, 2022, and December 3, 2022, and who had ≥30 days of continuous glucose monitor data, and ≥30% of closed-loop usage were included in the current analysis (N = 1805).

**Results:**

Time in range (3.9-10 mmol/L) was 72.6 ± 11.5% (mean ± SD) for all users and increased by age from 66.9 ± 11.7% for users ≤6 years old to 81.8 ± 8.7% for users ≥65 years. Time spent in hypoglycemia (<3.9 mmol/L) was 2.3% [1.3, 3.6] (median [interquartile range]). Mean glucose and glucose management indicator were 8.4 ± 1.1 mmol/L and 6.9%, respectively. Time using closed-loop was high at 94.7% [90.0, 96.9].

**Conclusions:**

Glycemic outcomes from the present real-world evidence are comparable to results obtained from previous randomized controlled studies and confirm the efficacy of this hybrid closed-loop system in real-world settings.

## Introduction

Closed-loop insulin delivery consists of an algorithm that utilizes real-time sensor glucose levels from a continuous glucose monitor (CGM) to direct insulin delivery via an insulin pump.^1^ Evidence from randomized controlled trials (RCTs) has shown that closed-loop insulin delivery can improve glycemic control in adults, accompanied by a reduction in the risk of hypoglycemia.^2^ In children and adolescents, similar improvements in glycemic control have been demonstrated, while not increasing the risk of hypoglycemia.^3^

RCTs tend to have strict inclusion and exclusion criteria, are conducted in “ideal” settings, and may not be generalizable to the broader population.^4^ Real-world data generated from real-world evidence (RWE) is important as it allows information to be collected in “real-world” settings on a larger number of individuals without imposing strict eligibility criteria.^4^ In the present analysis, we present RWE from non-study users of a hybrid closed-loop system across 15 countries and different age groups.

## Methods

### Design

We retrospectively analyzed data collected by a hybrid closed-loop system app between May 9, 2022, and December 3, 2022, from 15 countries (Australia, Austria, Czech Republic, Denmark, Finland, Germany, Ireland, Italy, Luxembourg, Netherlands, Poland, Spain, Sweden, Switzerland, and the United Kingdom). The following inclusion criteria applied: (1) users consented for their data to be analyzed, (2) users had 30 or more days of data available, and (3) users had at least 30% or more of closed-loop usage.

### mylife CamAPS FX Closed-Loop Insulin Delivery

In May 2022, the CamAPS FX app was integrated with mylife YpsoPump insulin pump (Ypsomed, Switzerland) and Dexcom G6 glucose sensor (Dexcom, CA, USA) and launched in several countries in Europe and Australia as mylife CamAPS FX. The adaptive model predictive control algorithm residing on the app automatically calculates the insulin infusion rate every 8 to 12 minutes, which is communicated wirelessly to the insulin pump.^1^ The closed-loop algorithm has a default target glucose level of 5.8 mmol/L, which is adjustable between 4.4 and 11.0 mmol/L across different times of day and night.

### Data Analysis and Statistical Methods

Analyses were performed for all users, as well as by age group and by country. Countries with at least 30 users were included in the latter analysis (Australia, Austria, Germany, Switzerland, and the United Kingdom). Year of birth was self-reported by users who agreed to share this information; users were classified into the following five age groups: ≤6, 7 to 14, 15 to 21, 22 to 64, and ≥65 years. The following endpoints were calculated and reported for the 24-hour period, as well as for the daytime (06:01 to 23:59) and the nighttime (00:00 to 06:00): mean sensor glucose, standard deviation (SD) and coefficient of variation (CV) of sensor glucose, time in target range between 3.9 and 10 mmol/L, time in hypoglycemia (<3.9 mmol/L), time with sensor glucose <3.0 mmol/L, and time with sensor glucose >10.0 mmol/L, and >13.9 mmol/L. Other reported metrics included glucose management indicator (GMI), time using closed-loop, and total daily insulin (units/day). Pre-closed-loop endpoints were not available as the app allows auto-mode to be initiated immediately following initialization. Glucose metrics were calculated using GStat software, version 2.3 (University of Cambridge, Cambridge, UK). Statistical analyses were conducted using R (version 4.2.3, R Foundation for Statistical Computing, Vienna, Austria). Normally distributed data are presented as mean ± SD and non-normally distributed data as median (interquartile range [IQR]).

## Results

**Figure 1.**
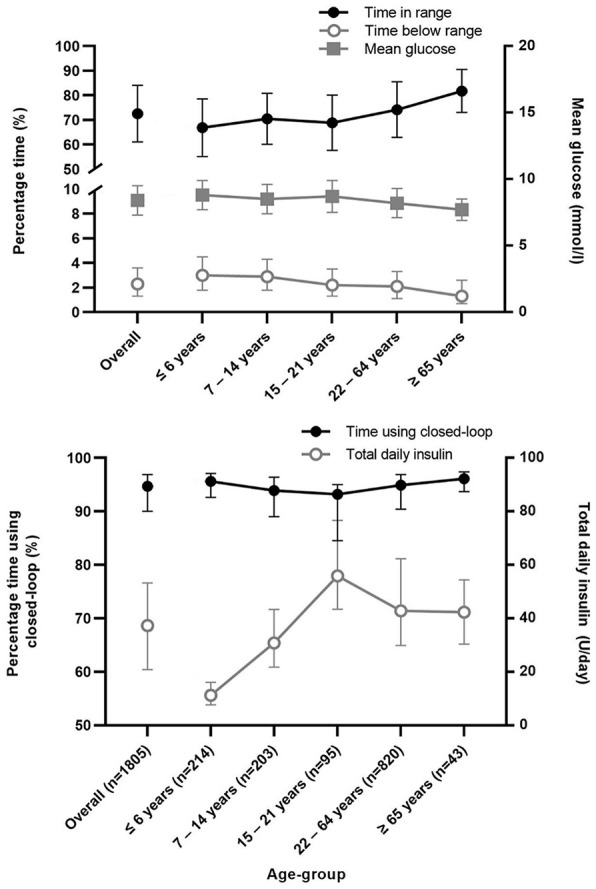
Top panel: Mean (standard deviation) percentage time with sensor glucose in target range, median (interquartile range) percentage time with sensor glucose below range, and mean glucose (standard deviation). Bottom panel: median (interquartile range) percentage time using closed-loop and total daily insulin.

A total of 1805 users from 15 countries who met the inclusion criteria were included in the present analysis. Glycemic and insulin metrics, overall and by age group, are presented in Table 1 and Figure 1. Mean age among all users was 30.2 ± 19.3 years (data on age was available for 1375 users). Time in range was 72.6 ± 11.5% (mean ± SD) for all users and rose gradually from 66.9 ± 11.7% for users aged less than 6 years to 81.8 ± 8.7% for users 65 years or older. Time in hypoglycemia (<3.9 mmol/L) was 2.3% (1.3-3.6) (median [IQR]) for all users and was greatest at 3.0% (1.8-4.5) for users aged less than 6 years and lowest at 1.3% (0.7-2.6) for users aged 65 years or older. A similar pattern was observed for time spent <3.0 mmol/L (all users 0.4% [0.2-0.7]). Mean time spent above 10 mmol/L was 24.7% ± 11.8 for all users and was greatest among the youngest age group and lowest for the oldest age group. Mean glucose was 8.4 ± 1.1 mmol/L (ranging from 7.7 ± 0.8 mmol/L to 8.8 ± 1.1 mmol/L across age groups) and mean GMI was 6.9%. Time using closed-loop was high across all age groups with a median of 94.7% (90.0-96.9).

**Table 1.** Characteristics and Glycemic and Insulin Outcomes of Users by Age Group.

	Overall	≤6 years	7-14 years	15-21 years	22-64 years	≥65 years
Users (n)	1805	214	203	95	820	43
Observation period (days)	84.0 (54.0, 118.0)	95.0 (61.0, 122.0)	84.0 (55.5, 117.0)	77.0 (47.5, 116.0)	88.0 (58.0, 124.0)	81.0 (59.5, 127.0)
Age (years)	30.2 ± 19.3	3.8 ± 1.5	10.3 ± 2.2	17.3 ± 2.0	41.4 ± 10.9	69.2 ± 3.4
Mean glucose (mmol/L)	8.4 ± 1.1	8.8 ± 1.1	8.5 ± 1.1	8.7 ± 1.2	8.2 ± 1.1	7.7 ± 0.8
Glucose SD (mmol/L)	3.1 ± 0.7	3.4 ± 0.7	3.3 ± 0.8	3.5 ± 0.9	2.9 ± 0.7	2.4 ± 0.5
Glucose CV (%)	36.2 ± 5.5	38.7 ± 4.5	38.9 ± 5.5	39.5 ± 5.9	35.1 ± 5.1	30.9 ± 4.1
GMI (%)	6.9	7.1	7.0	7.1	6.9	6.6
Percentage of time with glucose						
3.9-10.0 mmol/L	72.6 ± 11.5	66.9 ± 11.7	70.5 ± 10.4	68.9 ± 11.2	74.2 ± 11.3	81.8 ± 8.7
>10.0 mmol/L	24.7 ± 11.8	29.7 ± 12.0	26.3 ± 10.7	28.5 ± 11.5	23.3 ± 11.8	16.4 ± 9.1
>13.9 mmol/L	5.2 (2.5, 9.4)	7.9 (4.2, 13.4)	7.1 (3.9, 10.5)	8.6 (4.6, 13.7)	4.3 (1.9, 7.8)	1.8 (0.8, 3.2)
<3.9 mmol/L	2.3 (1.3, 3.6)	3.0 (1.8, 4.5)	2.9 (1.8, 4.3)	2.2 (1.3, 3.5)	2.1 (1.1, 3.3)	1.3 (0.7, 2.6)
<3.0 mmol/L	0.4 (0.2, 0.7)	0.5 (0.3, 0.9)	0.5 (0.3,0.9)	0.4 (0.2, 0.7)	0.3 (0.1, 0.6)	0.1 (0.1, 0.4)
Total daily insulin (U/day)	37.3 (20.8, 53.2)	11.2 (7.6, 16.0)	30.8 (21.7, 43.3)	55.9 (43.4, 76.6)	42.8 (29.9, 62.3)	42.3 (30.4, 54.4)
Time using closed-loop (%)	94.7 (90.0, 96.9)	95.6 (92.6,97.1)	93.9 (89.0, 96.4)	93.2 (84.5, 95.0)	94.9 (90.4, 96.9)	96.1 (93.7, 97.4)

Data are mean ± SD or median (IQR).

Abbreviations: CV; coefficient of variation; GMI; glucose management indicator.

Glycemic outcomes by country are displayed in Supplemental Table 1 (N = 1595). Time in range was highest among users in Australia at 76.3 ± 12.3% and Austria (76.0 ± 9.9%), followed by Germany, Switzerland, and the United Kingdom (67.2 ± 12.1%). Time in hypoglycemia (<3.9 mmol/L) was relatively low across all five countries. Mean time spent above 10 mmol/L was lowest in Austria (21.0 ± 9.9%) and Australia (21.3 ± 12.6%), and highest in the United Kingdom (29.7 ± 12.2%). Mean glucose was lowest in Austria and highest in the United Kingdom.

Glycemic outcomes by time of day (daytime versus nighttime) are shown in Supplemental Table 2. Time in range was higher in the nighttime (77.8 ± 12.7%) as compared with the daytime (70.8 ± 12.0%). Correspondingly, mean time spent in hypoglycemia (<3.9 mmol/L) was lower during the nighttime (1.7% [0.8%-2.9%]) and higher during the daytime (2.5% [1.4%-3.9%]), whereas mean time spent above 10 mmol/L was higher during the daytime (26.2 ± 12.4%) as compared with the nighttime (20.1 ± 12.6%). Similarly, mean glucose was higher during the daytime at 8.5 ± 1.2% as compared with the nighttime (8.0 ± 1.1%).

## Discussion

In this retrospective analysis of RWE data from 1805 users of a hybrid closed-loop system with type 1 diabetes, we found results similar to findings from previous RCTs confirming the efficacy of the closed-loop system in real-world settings.

Overall, mean time spent in range 3.9 to 10 mmol/L was at 72.6% and median time below 3.9 mmol/L was at 2.3%, in line with the glycemic targets recommended by international guidelines.^5,6^ Consistent with clinical studies, we found that users in the older age groups spent a higher time in target range and lower time in hypoglycemia as compared with the younger age groups.^7-9^ In a study involving adults aged 60 years or older,^8^ time spent in target range (79.9%) and time spent in hypoglycemia <3.9 mmol/L (1.7%) were similar to the current analysis in this particular age group. Likewise, a study carried out among adults older than 18 years found that time spent in target range was 75.0% and time spent in hypoglycemia <3.9 mmol/L was 2.9% (vs 74.2% and 2.1%, respectively, for adults aged 22-64 years in the present analysis).^9^

In a study conducted among very young children aged 1 to 7 years, time spent in target range and in hypoglycemia were 71.6% and 4.9%, respectively, which are comparable to our current findings.^7^ A recent trial performed in children aged 2 to 6 years demonstrated a mean time spent in target range of 64.2% and median time in hypoglycemia of 3.5%.^10^ RCTs have shown that it is challenging for children and adolescents to meet the time in range goal and tend to be at greater risk of hypoglycemia due to multiple factors including higher variability in insulin requirements and unpredictable patterns related to physical activity and meals.^3,11,12^ Our results highlight that children and adolescents face similar challenges in real-world settings.

Germany had the largest number of users. We found that, on average, users from Australia, Austria, and Germany were more likely to meet glycemic targets as compared with users from Switzerland and the United Kingdom. This may possibly be due to differences in baseline glucose control across participants from different countries, as it has been shown that baseline HbA1c is correlated with mean glucose during closed-loop use.^13^ Users had superior glycemic control during the nighttime as compared with the daytime with higher time in range and lower time spent in hypoglycemia. This is frequently observed in closed-loop studies^7,8^ as the majority of insulin delivered overnight is closed-loop driven (vs user-driven) and factors such as physical activity and meals are usually absent at night.^3^

Our findings are comparable to real-world data reported with the Tandem Control-IQ technology where 83% of users had T1D.^14^ The authors found that median percent time in range after 12 months of use of the closed-loop system was 73.6% among all users and that time in range increased from 64.7% among users 6 to 13 years of age to 79.0% among users aged 64 years or older.^14^ RWE from the Medtronic MiniMed 780G system showed that users aged 15 years or younger had a lower time in range (73.9%) as compared with users aged older than 15 years (76.5%).^15^ Recent real-world data from the Omnipod DASH system demonstrate an improvement in HbA1c after 3 months of use (a reduction in baseline HbA1c of 0.9 ± 1.6 in adults and 0.9 ± 2.0% in the pediatric cohort), with a simultaneous reduction in hypoglycemic events.^16^

To our knowledge, this is the first RWE analysis of data of a hybrid closed-loop system that includes very young children (≤6 years old). Other strengths are the use of real-world data with only a few selection criteria, as well as the reporting of results across different age groups. The limitations of this analysis include the retrospective nature of the study, the lack of demographic data (other than year

of birth), medical history, HbA1c data, and absence of data before activation of the closed-loop system. A possible bias related to “early adopters” of the closed-loop system cannot be excluded.

## Conclusions

We conclude that glycemic outcomes from the present RWE analysis are comparable to results obtained from our previous randomized studies and demonstrate that users, on average, were able to achieve glycemic targets as outlined by international guidelines. Our results also highlight the high closed-loop usage among users in real-world free-living settings.

## Supplemental Material

sj-docx-1-dst-10.1177_19322968231185348 – Supplemental material for Real-World Evidence Analysis of a Hybrid Closed-Loop System Supplemental material, sj-docx-1-dst-10.1177_19322968231185348 for Real-World Evidence Analysis of a Hybrid Closed-Loop System by Heba Alwan, Malgorzata E. Wilinska, Yue Ruan, Julien Da Silva and Roman Hovorka in Journal of Diabetes Science and Technology
