# Supplementary material for: Real-World Evidence Analysis of a Hybrid Closed-Loop System
Source: J Diabetes Sci Technol. 2023 Jul 8;19(2):385–9. doi: 10.1177/19322968231185348 (PMC11873883; doi:10.1177/19322968231185348)
Supplement: sj-docx-1-dst-10.1177_19322968231185348 – Supplemental material for Real-World Evidence Analysis of a Hybrid Closed-Loop System [file sj-docx-1-dst-10.1177_19322968231185348.docx]

**Supplemental Table 1. Characteristics and glycemic outcomes of mylife CamAPS FX users by country**

|  |  | **Germany** | **Austria** | **UK** | **Switzerland** | **Australia** |
| --- | --- | --- | --- | --- | --- | --- |
| Users (n) | | 1,051 | 142 | 141 | 139 | 122 |
| Age (years) | | 30.3±19.6 | 26.8±18.7 | 23.6±18.4 | 32.2±19.0 | 37.2±17.0 |
| Mean glucose (mmol/L) | | 8.3±1.0 | 8.0±0.9 | 8.8±1.2 | 8.6±1.4 | 8.1±1.2 |
| Glucose SD (mmol/L) | | 3.1±0.7 | 2.9±0.7 | 3.4±0.8 | 3.2±0.9 | 2.8±0.8 |
| Glucose CV (%) | | 36.3±5.3 | 35.7±5.6 | 38.5±5.9 | 36.2±6.2 | 34.5±5.5 |
| Percentage of time with glucose | |  |  |  |  |  |
|  | 3.9-10.0 mmol/L | 72.8±10.6 | 76.0±9.9 | 67.2±12.1 | 69.8±14.1 | 76.3±12.3 |
|  | >10.0 mmol/L | 24.5±10.9 | 21.0±9.9 | 29.7±12.2 | 27.7±14.4 | 21.3±12.6 |
|  | >13.9 mmol/L | 5.2 (2.7, 9.0) | 4.0 (1.9, 7.4) | 7.9 (4.3, 13.6) | 5.7 (3.0, 12.0) | 3.7 (1.4, 6.8) |
|  | <3.9 mmol/L | 2.3 (1.3, 3.5) | 2.5 (1.4, 3.9) | 2.5 (1.6, 4.2) | 2.2 (1.1, 3.4) | 2.1 (1.1, 3.2) |
|  | <3.0 mmol/L | 0.4 (0.2, 0.7) | 0.4 (0.2, 0.7) | 0.5 (0.2, 0.9) | 0.4 (0.2, 0.7) | 0.4 (0.1, 0.6) |

Data are mean±SD or median (IQR).

SD=standard deviation. CV=coefficient of variation.

Only countries with 30 or more users were included in the present analysis (N=1,595).

**Supplemental Table 2. Characteristics and glycemic outcomes of mylife CamAPS FX users by time-of-day**

|  |  | **Daytime*** | **Nighttime*** |
| --- | --- | --- | --- |
| Mean glucose (mmol/L) | | 8.5±1.2 | 8.0±1.1 |
| Glucose SD (mmol/L) | | 3.1±0.8 | 2.7±0.8 |
| Glucose CV (%) | | 36.6±5.5 | 33.4±6.6 |
| Percentage of time with glucose | |  |  |
|  | 3.9-10.0 mmol/L | 70.8±12.0 | 77.8±12.7 |
|  | >10.0 mmol/L | 26.2±12.4 | 20.1±12.6 |
|  | >13.9 mmol/L | 5.6 (2.7, 10.3) | 3.3 (1.2, 6.8) |
|  | <3.9 mmol/L | 2.5 (1.4, 3.9) | 1.7 (0.8, 2.9) |
|  | <3.0 mmol/L | 0.4 (0.2, 0.7) | 0.3 (0.1, 0.6) |

Data are mean±SD or median (IQR).

SD=standard deviation. CV=coefficient of variation.

All users who consented to have their data analyzed were included in this analysis (N=1,805).

*Daytime is from 06:01 to 23:59, nighttime is from 00:00 to 06:00.
